# Correction: A hierarchical integrated 3D carbon electrode derived from gingko leaves *via* hydrothermal carbonization of H_3_PO_4_ for high-performance supercapacitors

**DOI:** 10.1039/d3na90113k

**Published:** 2023-11-27

**Authors:** Han Liu, Fumin Zhang, Xinyu Lin, Jinggao Wu, Jing Huang

**Affiliations:** a State Key Laboratory of Silkworm Genome Biology, College of Sericulture, Textile and Biomass Sciences, Westa College, Southwest University Chongqing 400715 PR China hj41012@163.com; b Key Laboratory of Rare Earth Optoelectronic Materials & Devices, College of Chemistry and Materials Engineering, Huaihua University Huaihua 418000 PR China

## Abstract

Correction for ‘A hierarchical integrated 3D carbon electrode derived from gingko leaves *via* hydrothermal carbonization of H_3_PO_4_ for high-performance supercapacitors’ by Han Liu *et al.*, *Nanoscale Adv.*, 2023, **5**, 786–795, DOI: https://doi.org/10.1039/D2NA00758D.

The authors regret that the acknowledgement of College Student Innovation and Entrepreneurship Training Program, Southwest University (S202210635259) is incorrect. The correct acknowledgement is College Student Innovation and Entrepreneurship Training Program, Southwest University (S202310635215).

The Royal Society of Chemistry apologises for these errors and any consequent inconvenience to authors and readers.

## Supplementary Material

